# Operando Ru and Ti K‐Edge X‐Ray Absorption Study of the Low‐Temperature Sabatier Reaction on Ru/TiO_2_ Catalysts

**DOI:** 10.1002/cphc.202500397

**Published:** 2025-12-17

**Authors:** Joachim Bansmann, Shilong Chen, Ali M. Abdel‐Mageed, R. Jürgen Behm

**Affiliations:** ^1^ Institute of Surface Chemistry and Catalysis/Institute of Physical Chemistry II Ulm University D‐89069 Ulm Germany; ^2^ Leibniz‐Institute for Catalysis (LIKAT) D‐18059 Rostock Germany; ^3^ Institute of Theoretical Chemistry Ulm University D‐89069 Ulm Germany; ^4^ Present address: National Engineering Research Center of Chemical Fertilizer Catalyst School of Chemical Engineering Fuzhou University 350002 Fuzhou P. R. China

**Keywords:** CO_2_ methanation, extended fine structure X‐ray absorption spectroscopy, operando spectroscopy, Ru/TiO_2_ catalyst, X‐ray absorption near edge structure spectroscopy, X‐ray absorption spectroscopy

## Abstract

Stimulated by recent findings of a beneficial effect of a high‐temperature treatment on the activity and selectivity of highly active and selective Ru/TiO_2_ catalysts in the CO_*x*_ methanation, a detailed study of the dynamic changes in the chemical and structural properties is performed, induced by this treatment and their correlation with the changes in the catalytic performance of the catalyst. These changes are characterized by time‐resolved operando X‐ray absorption spectroscopy at the Ru and Ti K‐edges, together with structural characterization by high‐resolution transmission electron microscopy. The observation of differently long times required for the reduction of the oxidic Ru nanoparticles in CO‐free CO_2_/H_2_ gas mixtures (1000 min) and in trace amounts of CO containing CO/CO_2_/H_2_ gas mixtures (100 min) under reaction conditions (190 °C, atmospheric pressure) correlates very well with the different times required for catalyst activation in these reaction gas mixtures.

## Graphical abstract

Operando X‐ray absorption spectroscopy (XAS) was used to characterize Ru/TiO_2_ nanoparticle catalysts in the CO_x_ methanation reaction. Near‐edge XAS at the Ru K‐edge offers access to the oxidation state of the Ru nanoparticles at different reaction stages, the Ti K‐pre‐edge region provides information on the number of O‐vacancies/the oxidation state of the support. Structural information is obtained from the extended fine structure.

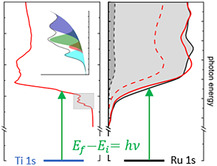

## 1 Introduction

The reduction of carbon dioxide to methane or other C1 molecules using “green hydrogen”, i.e., hydrogen produced via electrolysis of water with electric energy from renewable sources, has attracted increasing interest as a promising route for massive energy storage in renewable energy concepts (chemical energy storage)^[^ ^1^, ^2^, ^3^, ^4^ ^]^ or, alternatively, as first step for the synthesis of hydrocarbon based fuels (solar fuels) or chemicals.^[^ ^5^, ^6^, ^7^ ^]^ Equally important, methane and other C1 molecules are easily transportable in existing infrastructures, an important aspect for promoting the introduction of renewable energies into the market.^[^ ^8^ ^]^

Supported Ru catalysts were intensively studied in recent years in CO/CO_2_/H_2_ methanation reactions because they are considered to be among the most active candidates for these reactions at relatively mild conditions (temperatures from 150 to 200 °C, ambient pressure).^[^ ^9^, ^10^, ^11^, ^12^ ^]^ Further studies had indicated that the activity and selectivity of these catalysts depend sensitively also on the catalyst pretreatment. For instance, we had demonstrated that the activity and selectivity of a Ru/TiO_2_ catalyst in CO_*x*_ hydrogenation reactions can be tuned and further improved by a higher‐temperature treatment in the reaction atmosphere, by a temperature programed reaction (TPR) treatment.^[^ ^11^, ^13^, ^14^ ^]^ We had attributed this improved performance to an increase in the local charge density on the Ru nanoparticles (NPs) due to charge transfer from *O*‐vacancy defects to the Ru nanoparticles, more specifically, to the interface Ru atoms of the nanoparticles, together with a partial encapsulation of the Ru NPs by a TiO_*x*_ layer.^[^ ^14^ ^]^ These changes depended strongly on the specific surface area of the support, at about constant Ru particle size and Ru loading.^[^ ^14^ ^]^ While catalysts with small specific surface area/large TiO_2_ particle size are 100% selective for CO_2_ methanation during reaction at 190 °C, both before and after the TPR procedure, catalysts with high specific surface area/small TiO_2_ particle were found to change their selectivity from 100% CO_2_ methanation before the TPR procedure to 100% CO formation after the TPR procedure. Based on a systematic characterization of the catalysts before and after the TPR procedure, which revealed distinct changes in their structure, oxidation state and electronic state, we proposed that the chemical modification of the support by O‐vacancy formation leads to an electronic modification of the interface perimeter Ru sites,^[^ ^15^, ^16^ ^]^ which in turn affects the reaction behavior and selectivity of these sites in the CO_2_ reduction reaction. Modest reduction of the support enhances the CO_2_ methanation activity, while over‐reduction changes the selectivity from 100% methanation to 100% CO formation via the reverse water–gas shift (RWGS) reaction for Ru/TiO_2_ catalysts based on high specif‐surface area (>200 m^2^ g^−1^).^[^ ^14^ ^]^

While these changes in the reaction behavior and the underlying physical reasons seem to be well understood, much less is known on the dynamics of the structural and chemical changes of the catalyst during reaction in a CO_2_/H_2_ gas mixture (15.5% CO_2_, 3% N_2_, balance H_2_). This is topic of the present study, where we followed dynamic changes of a Ru/TiO_2_ catalyst during the reaction at the standard reaction temperature of 190 °C and during a subsequent TPR treatment by time‐resolved operando X‐ray absorption spectroscopy (XAS). The dynamic changes in the chemical and structural properties are compared with changes in the reaction behavior. We are particularly interested in correlations between these properties.

Experimental details in the method section (Section 4). We start with a compilation of time‐resolved kinetic data that characterize the dynamic reaction behavior during reaction in CO_2_/H_2_ mixtures with either no or small amounts of CO (0.6 vol%) at 190 °C and during the TPR treatment (Section 2.1), changes in the electronic state of the catalyst surface, specifically in the oxidation state of the Ru species, are evaluated from time‐resolved operando X‐ray absorption near‐edge structure spectroscopy (XANES) measurements at the Ru K‐edge (section 2.2). To directly track changes in the electronic properties of the catalyst support, we also performed similar measurements at the Ti K‐edge (section 2.2). Possible changes in the Ru particle size and shape are identified by operando extended fine structure X‐ray absorption spectroscopy (EXAFS) and by high‐resolution (scanning) transmission electron microscopy measurements (section 2.3). Finally, we briefly summarize the main findings on the catalyst activation and deactivation processes during time on stream and during the TPR treatment, comparing also with Ru nanoparticle catalysts supported on other oxides (section 3).

## 2 Results

### 2.1 Summary of Relevant CO_*X*_ Methanation Kinetics

**Figure 1:**
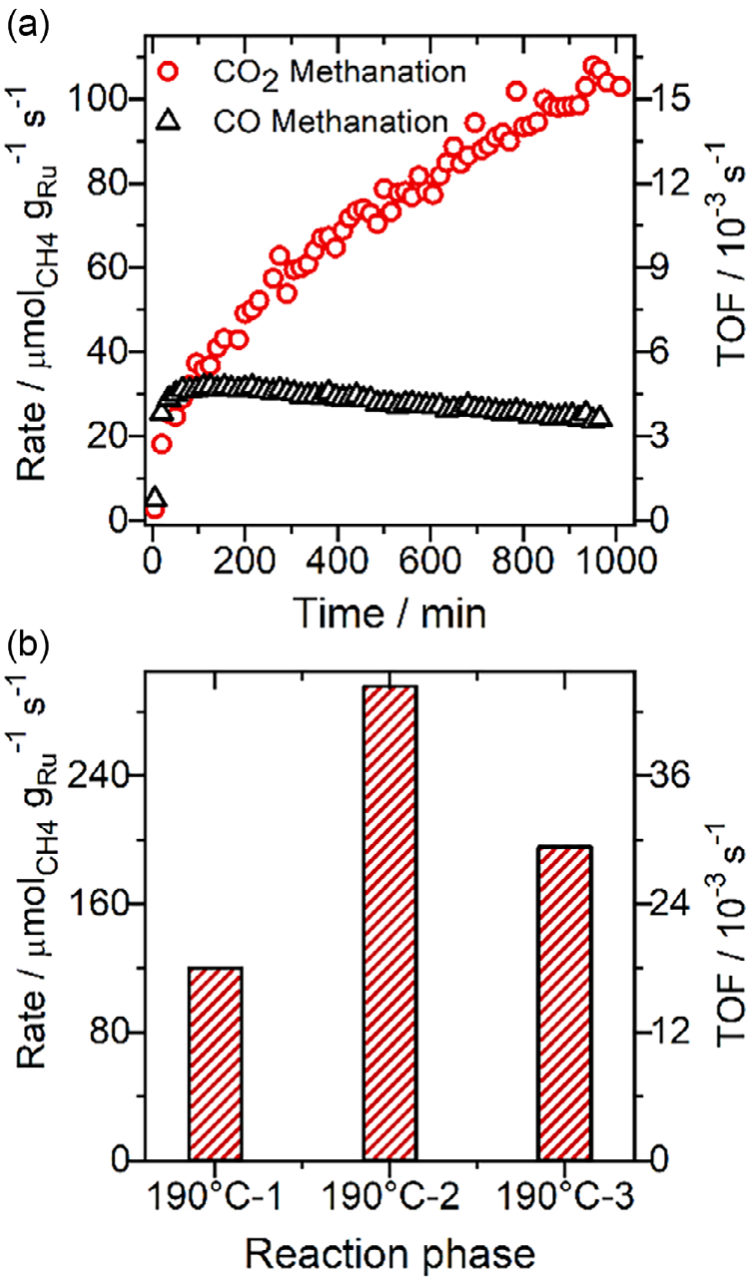
a) Ru‐mass‐normalized methane formation rate (left *y*‐axis) and the corresponding turnover frequency (right *y*‐axis) during CO_2_ methanation in CO_2_‐ref (15.5% CO_2_, 3% N_2_, balance H_2_) and during CO methanation in SR‐ref 6000 (0.6% CO, 15.5% CO_2_, 3% N_2_, balance H_2_) gas mixtures at 190 °C after calcination (O150: 30 min in 10% O_2_/N_2_ at 150 °C) of the catalyst. b) Ru‐mass‐normalized methane formation rate (left)/TOF (right) from CO_2_ in CO_2_‐ref at 190 °C: i) at the end of the 190 °C‐1 phase, ii) at the end of a 190 °C phase after the subsequent TPR sequence (190 °C‐2), and iii) after recalcination (30 min in 10% O_2_/N_2_ at 150 °C) and after 3 h on stream in CO_2_‐ref reformate (190 °C‐3).

**Figure 2:**
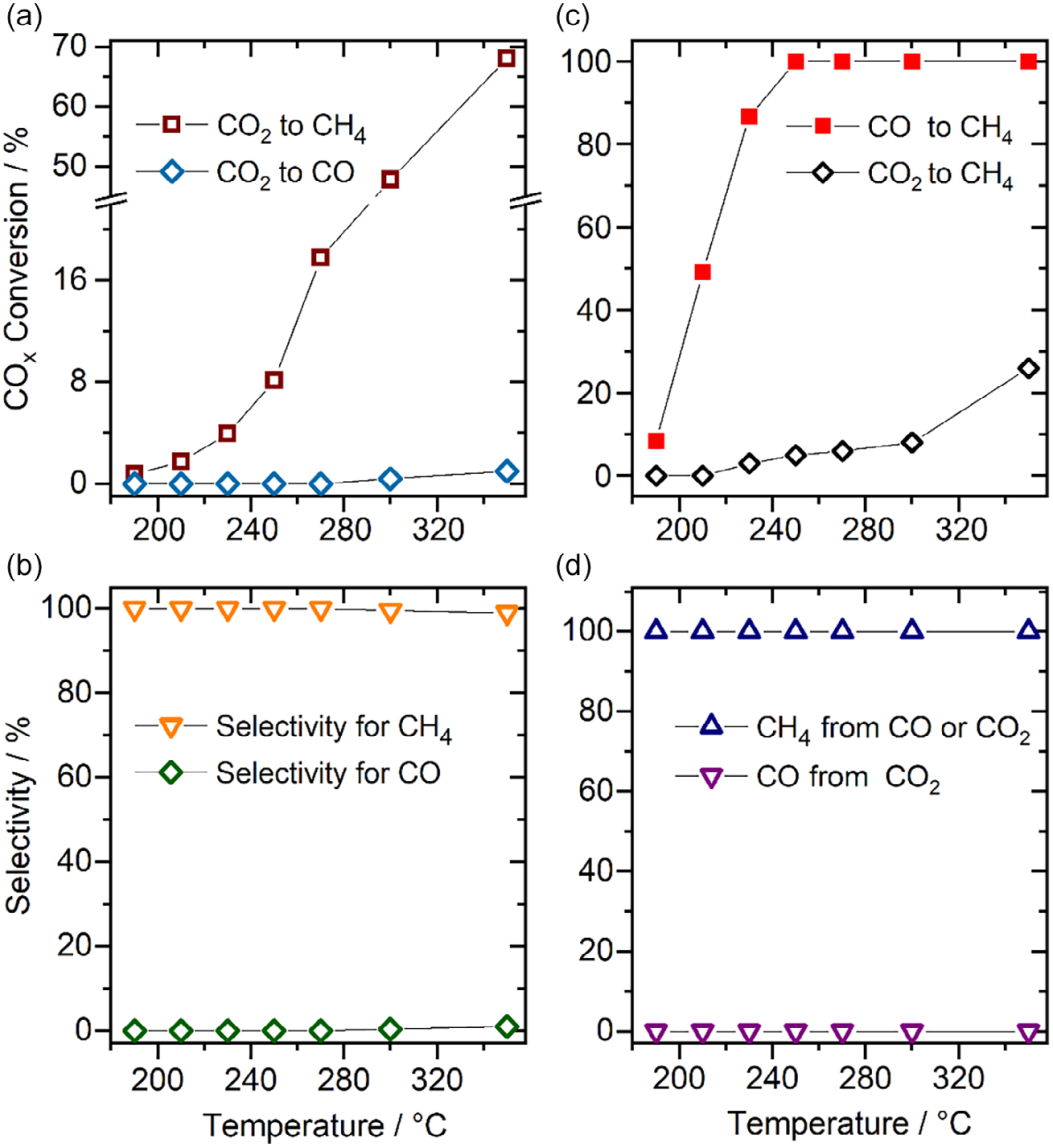
a) Temperature‐dependent CO_2_ conversion to methane and CO in CO_2_‐ref reformate and b) the corresponding selectivity for methane and CO in CO_2_‐ref reformate at different temperatures. c) Temperature‐dependent conversion of CO_2_ and CO to methane in CO and CO_2_ containing SR‐ref 6000 reformate and d) the corresponding selectivity for methane and CO formation in SR‐ref 6000 reformate at different temperatures.

Before presenting the operando XAS data, we will briefly summarize results of kinetic measurements relevant for this study, which had partly been reported earlier.^[^ ^13^, ^14^, ^17^ ^]^ For reaction in a CO_2_/H_2_ mixture (CO_2_‐ref), with no CO in the reaction gas mixture, the conversion to CH_4_ increases steadily with time for reaction at 190 °C over 1000 min on stream (**Figure** **1**a), with no indication of CO formation via the RWGS reaction (**Figure** **2**a). The reaction rate is indicated by the left bar (190 °C‐1) in Figure 1b.

Here, it should be noted that in this measurement, we can only determine the effective CO formation and selectivity for CO formation under flow conditions, which means that we cannot rule out that there is some CO formation from CO_2_, which in a second step is immediately converted into CH_4_. During a subsequent stepwise TPR sequence up to 350 °C, the CH_4_ formation increased steeply (Figure 2a). Only at the highest temperatures, between 300 and 350 °C, measurable CO formation was observed (Figure 2a). This results in a selectivity for methane formation of 100% at lower temperatures, and of >99% at 350 °C (Figure 2b). Most importantly, after cooling the catalyst to the initial temperature of 190 °C (190 °C‐2), the CO_2_ conversion and thus the CO_2_ methanation rate are about 2.5 times higher than before the TPR sequence (190 °C‐1) (Figure 1b). The selectivity for methane formation remains at 100%.

A test of the reversibility of these changes, by exposing the catalyst to a second calcination step (10% O_2_/N_2_ for 1 h at 190 °C) after the 190 °C‐2 reaction phase, followed by another reaction phase (3 h) at 190 °C (190 °C‐3), revealed a significant loss in activity. The resulting reaction rate of 195 μmol_CH4_ g_Ru_^−1^ s^−1^ (TOF: 28.5 × 10^−3^ s^−1^) (Figure 1b) is clearly lower than that during the 190 °C‐2 reaction phase, although still significantly higher than that before the TPR sequence (190 °C‐1). Apparently, the TPR treatment was not fully reversible under these conditions.

Similar measurements were also performed in a SR‐ref 6000 gas mixture (15.5% CO_2_, 0.6% CO, 80.9% H_2_, N_2_ balance) (Figure 1a, black symbols).^[^ ^15^, ^17^ ^]^ Under these conditions, in the presence of relatively small amounts of CO in the CO_2_/H_2_ gas mixture, the catalyst was 100% selective for methane formation, and this resulted entirely from the methanation of CO rather than of CO_2_.^[^ ^17^ ^]^ Also in this measurement, we refer to the effective selectivity for CO formation, which was determined from possible changes in the CO concentration. Different from the behavior during CO_2_ methanation in CO_2_‐ref, the catalyst showed only a rather short initial activation phase of about 120 min, where the rate increased from 5.2 μmol_CH4_ g_Ru_^−1^ s^−1^ after 10 min on stream to a maximum value of ≈32 μmol_CH4_ g_Ru_^−1^ s^−1^ after ≈120 min (Figure 1a).^[^ ^17^ ^]^ This was followed by a continuous slow deactivation, with the CO methanation rate decaying to about 24 μmol_CH4_ g_Ru_^−1^ s^−1^ after 1000 min on stream.^[^ ^15^, ^17^ ^]^ The inhibition of the CO_2_ methanation in this reaction mixture was previously explained by Ru surface site blocking effects, where adsorbed CO inhibits the adsorption of CO_2_ and thus also its further reaction on Ru or at the Ru‐TiO_2_ interface perimeter sites.^[^ ^17^, ^18^, ^19^ ^]^

During a TPR sequence in SR‐ref 6000 (190 to 350 °C), full CO conversion to methane was reached at 250 °C. At slightly lower temperature (230 °C), CO_2_ methanation started and increased with temperature, reaching a conversion of ≈27% at 350 °C. Also, during the TPR sequence, the effective selectivity for methane formation remained at 100% (no CO formation via the reverse water–gas shift (RWGS) reaction) (Figure 2e). Interestingly, the increase in CO_2_ conversion to methane during the TPR sequence in SR‐ref 6000 was significantly smaller than that observed in CO_2_‐ref reformate gas, in the absence of CO. Obviously, even the very small amount of CO has a pronounced effect on the methanation of CO_2_ on the Ru/TiO_2_ catalyst under these conditions. These results were in good agreement with the trends in previous reports on this reaction over a similar Ru/TiO_2_ catalyst^[^ ^13^ ^]^ and over a Ru/TiO_2_ catalyst with a lower surface area support (P25, Degussa, 60 m^2^ g^−1^),^[^ ^11^ ^]^ respectively.

In total, these data clearly demonstrated that i) even small amounts of CO in a CO_2_/H_2_ reaction gas mixture lead to pronounced changes in the activation and deactivation behavior of the Ru/TiO_2_ catalyst at 190 °C, and that ii) the TPR treatment results in a significant increase of the activity for CO_2_ methanation in a CO_2_/H_2_ mixture at 190 °C, and that iii) the TPR‐induced structural or electronic changes persist even upon reoxidation of the catalyst at 150 °C.

### 2.2 Reaction Induced Changes in the Electronic Properties

**Figure 3:**
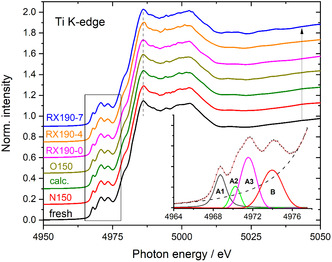
XANES spectra of the Ru/TiO_2_ catalysts recorded at the Ti K‐edge at different stages during pretreatment (N150, calc., O150, see text) and after different times on stream during reaction in CO_2_‐ref reformate (see text) as indicated in the figure. The dashed vertical line indicates the constant position of the maximum of the pre‐edge region. Inset: pre‐edge region of the N150 spectrum with a fit based on 4 peaks (peaks see figure, dashed line: background intensity).

In this section, we focused on changes in the electronic properties and, in particular, in the oxidation state of the Ru and Ti species, which were explored by operando XANES measurements at the Ru and Ti K‐edges. In a first step, we evaluate changes at the Ti K‐edge during pretreatment and reaction in CO_2_‐ref, which provide information on modifications in the electronic state/oxidation state of the Ti^n+^ ions. Spectra recorded at different stages before and during the reaction are shown in **Figure** **3**. Starting with a spectrum of the fresh catalyst at room temperature (RT, fresh), this is followed by spectra recorded at 150 °C in N_2_ (N150), during calcination in 10% O_2_/N_2_ for 30 min at 150 °C (calc.), after calcination while purging at 150 °C in N_2_ (O150), and at different representative times during the reaction at 190 °C (RX190‐0: first reaction spectrum, RX190‐4: spectrum started after 2 h, and RX190‐7: spectrum started after 4.5 h (last spectrum)). All spectra were normalized to the same intensity at the Ti K‐edge. The dashed vertical line indicates the constant position of the maximum of the pre‐edge region. Although on a first glance, all spectra look more or less identical, there are some changes in the pre‐edge region (see the rectangular box in the left part of the figure), which exhibit characteristic peaks. They are better visible in the inset in the bottom right part of Figure 3, which shows an enlarged representation of this region with a polynomial fit of the background (dashed line) and the individual peaks with their typical notation (A1‐A3, B). In the following, we will concentrate on the pre‐edge region of these spectra.

The pre‐edge peaks were previously assigned to electronic transitions from the Ti 1s orbital into Ti 3d and O 2p states.^[^ ^20^ ^]^ Other authors attributed the peaks A1–A3 to transitions into hybridized Ti 3d–4p states, whereas peak B was assigned to a transition into hybridized Ti 4p–4s states.^[^ ^21^, ^22^, ^23^ ^]^ Specifically, the A2 peak was attributed to fivefold coordinated Ti_5c_^4+^ species and thus to the presence of *O*‐vacancy defects at the surface of anatase nanocrystals.^[^ ^24^, ^25^, ^26^, ^27^ ^]^

**Figure 4:**
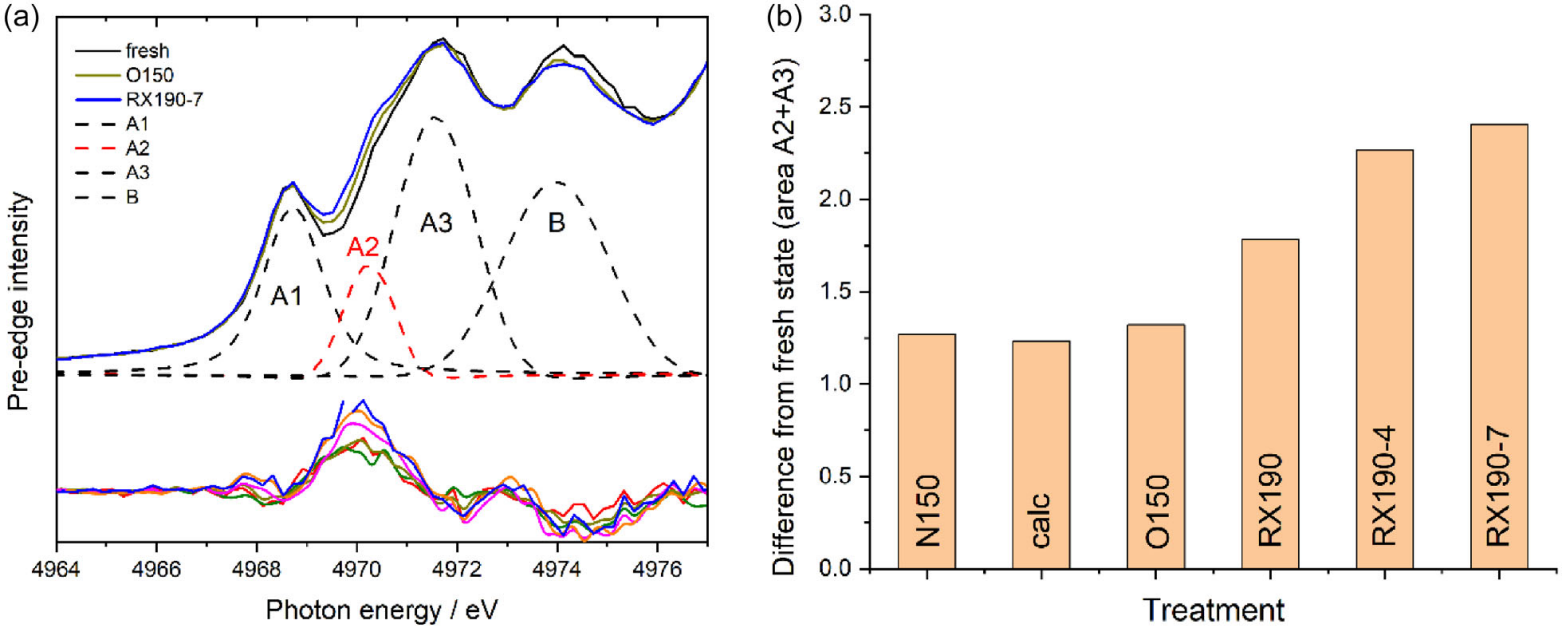
a) Upper part: background corrected XANES spectra recorded at different stages during pretreatment/reaction in CO_2_‐ref reformate at 150 °C (black: fresh catalyst in N_2_, dark yellow: during purging in N_2_ at 150 °C after calcination (O150), blue: during reaction after 4.5 h on stream (RX 190‐7). Lower part: enlarged difference spectra referenced to the fresh catalyst (red: N150; olive: calc., dark yellow: O150, magenta: RX190‐0, orange: RX190‐4, blue: RX190‐7). b) Variation of the combined intensity (in arb. units) of peaks A2 and A3 during the sequence indicated in Figure 3.

The pre‐edge features were evaluated by fitting the four peaks A1–A3 and B after background subtraction with Pseudo–Voigt peaks (see inset in Figure 3). Background‐subtracted spectra of the pre‐edge region recorded on the fresh catalysts (black), during purging in N_2_ at 150 °C after calcination (O150, magenta), and at the end of the reaction at 190 °C (RX190‐7: blue) are presented in the upper panel of **Figure** **4**a. The rather small differences between the spectra recorded at different stages of the pretreatment and the reaction and that of the fresh catalyst, enlarged by a factor 2, are displayed in the lower part of Figure 4a. The largest differences appear in the region of the pre‐edge peaks A2 and A3, whereas only minor changes are present in the areas of the A1 and B peak (cf. Figure 4a, upper panel). This is in good agreement with the assignment that the A1 and B peaks are due to transitions in Ti_6C_^4+^ species, whose relative concentration does not change markedly during the entire sequence. Considering that the area of peak A2 increases with the content of O vacancies, annealing of the fresh catalyst in N_2_ at 150 °C results in a small increase in the O vacancy concentration (Figure 4a, lower panel). This does not change measurably during subsequent calcination in 10% O_2_/N_2_ at 150 °C and also not during a subsequent purging in N_2_ at this temperature (Figure 4a, lower panel). Hence, in total, this sequence causes a slight reduction of the catalyst support compared to the fresh state. Similar effects have been observed earlier on different pure and Si‐doped Ru/TiO_2_ catalysts in respective treatments and discussed in detail.^[^ ^28^ ^]^ The subsequent exposure to the reaction atmosphere at 190 °C causes a distinct increase in the A2 peak, indicative of a substantial increase in the O vacancy surface concentration. Hence, the ongoing chemical reaction at 190 °C causes an ongoing increase in the O vacancy concentration, which decreases, however, toward the end of the reaction (cf. RX190‐7 in Figure 4a, lower panel).

This trend in O‐vacancy concentration during the different pretreatment and reaction steps is illustrated quantitatively in Figure 4b, where we plotted the intensity differences between the peaks A2 + A3 during the different pretreatment and reaction steps, as indicated in Figure 3, and that of the initial fresh catalyst. The peaks A2 and A3 were not separated because of their large overlap.

**Figure 5:**
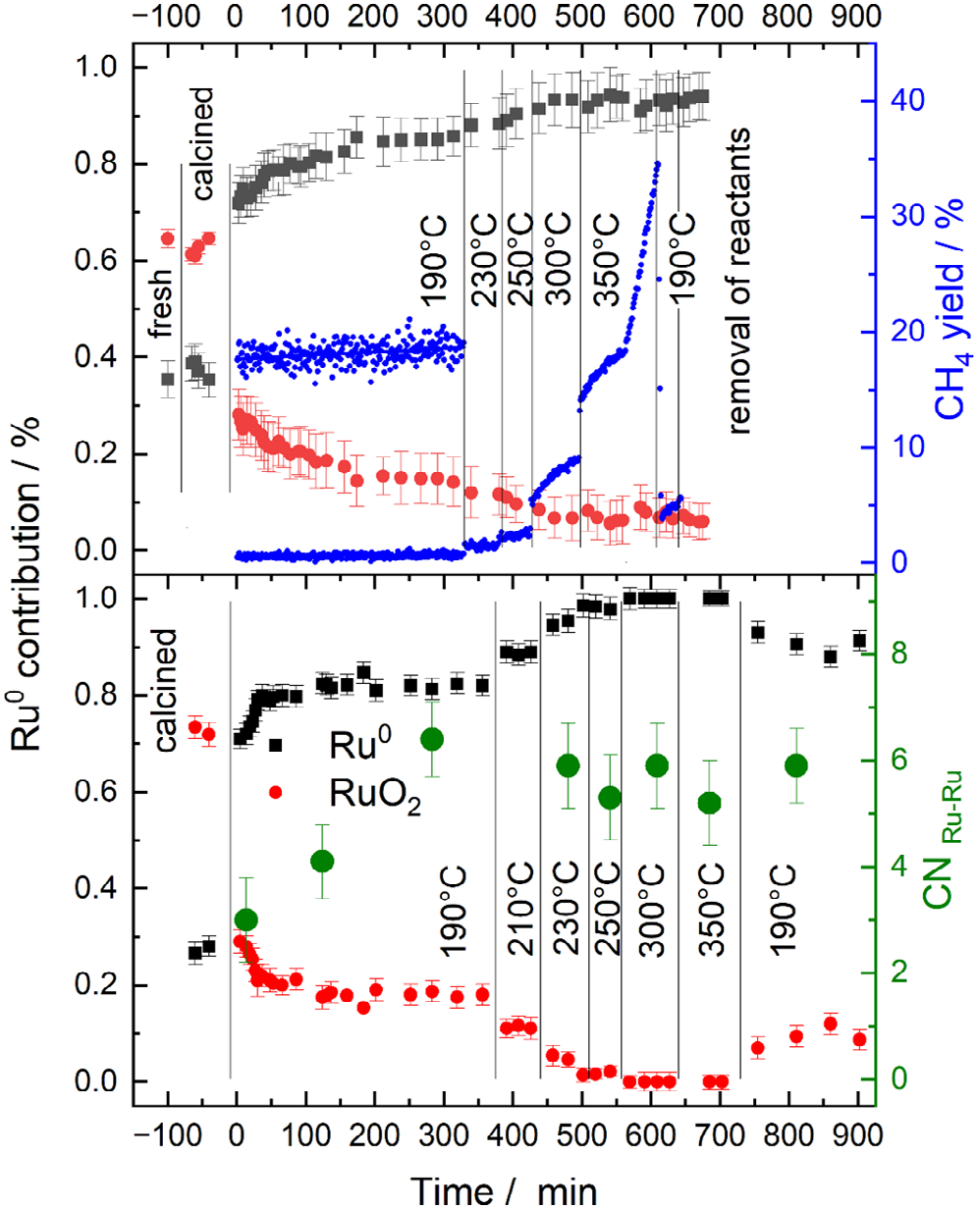
Relative contribution of Ru^0^ and RuO_2_‐like species in the Ru/TiO_2_ catalyst during the pretreatment and the reaction sequence in different reformate gas mixtures indicated in the Figure (see also Figure 1 and 2), as derived from an LC analysis of the XANES spectra. Upper panel: pretreatment and reaction sequence in CO_2_‐ref, lower panel: pretreatment and reaction in SR‐ref 6000 reformate, both as indicated in Figure 1 and 2. The scales on the right *y*‐axes indicate the CH_4_ yield measured simultaneously in the top panel and the coordination number CN_Ru−Ru_ in the lower panel as derived from the EXAFS data in Figure 6.

In the next step, we concentrate on the chemical nature of the Ru species during the pretreatment and reaction sequence, employing operando XANES measurements at the Ru K‐edge. The fraction of metallic and oxidic Ru species was determined by linear combination analysis (LCA), using appropriate Ru references (Ru foil, RuO_2_ powder, and RuCl_3_).^[^ ^17^, ^29^, ^30^ ^]^ Representative spectra recorded in CO_2_‐ref reformate are presented in Figure S1, in the Supporting Informtion, raw data in Figure S2, in the Supporting Information, and those in the SR‐ref 6000 reformate in Figure S3 and S4, Supporting Information. **Figure** **5** displays the evolution of the fractions of metallic and oxidic Ru species (black and red symbols) during the complete reaction sequence (see Figure 1), starting from the fresh catalyst in N_2_ at RT via the calcination pretreatment, the CO_2_ methanation reaction period in CO_2_‐ref (190 °C‐1), the TPR procedure (stepwise temperature increase from 190 to 350 °C in the reaction gas mixture) and finally a spectrum recorded during reaction at 190 °C in CO_2_‐ref (190 °C‐2), after cool‐down from 350 to 190 °C. The different temperatures are indicated in the figure. The last measurement was performed after removal of the reactants, upon changing to a N_2_ atmosphere at 190 °C. Additionally, the diagram also shows the methane conversion in the XAS reactor (blue dots, related scale at the right side in blue), which was recorded in parallel to the XAS measurements. The native state of the catalyst is characterized by a high contribution of RuO_2_‐like oxidic species, and this chemical state remains also during the calcination process at 150 °C in 10% O_2_/N_2_. With the onset of the CO_2_ methanation reaction at 190 °C in the reducing atmosphere (period 190 °C‐1), the Ru oxidation state changes rapidly to a more metallic state with initially ≈70% of metallic Ru. This further increases during the first 3 h on stream to about 85% of metallic Ru, and then stays more or less constant. Upon raising the temperature, the content of metallic Ru^0^ further increases to slightly above 90%, and it more or less remains at that level upon returning to 190 °C under reaction conditions. Hence, there is no obvious reoxidation by reaction with CO_2_ upon lowering the temperature or upon subsequent removal of the reactants, returning to a N_2_ atmosphere.

The simultaneously measured formation of CH_4_ (CO_2_ conversion) shows a small increase during reaction at 190 °C (not visible on this scale), followed by a steep increase in the conversion during the TPR procedure. Upon returning to 190 °C (190 °C‐2), it drops again. But similar to the observation during the kinetic measurements, the conversion is higher after the TPR procedure than before. Although, because of the different reaction conditions, the conversions and also their variation in time cannot be directly compared with the data acquired in the micro‐reactor (Figure 1a), the trends are comparable.

Following the approach used in the kinetic measurements, we performed a similar experiment in a SR‐ref 6000 atmosphere reaction mixture with trace amounts of CO. Here, we are particularly interested in identifying possible differences in the activation/deactivation phase, as they were indicated in the kinetic data in Figure 1and 2. Evaluation of the XANES spectra via a similar LC analysis results in the graph shown in the bottom panel in Figure 5. While the general trends are comparable to those obtained in the CO_2_‐ref reaction gas mixture (Figure 5, upper panel), a more detailed inspection reveals some distinct differences. First, the reduction of the RuO_*x*_ catalyst during reaction at 190 °C is much faster than in the CO_2_‐ref mixture. The final content of above 80% Ru^0^ content is reached already after 100 min, and then it remains about constant. Second, the increase of the metallic Ru^0^ content during the TPR sequence is more pronounced than in the CO_2_‐ref mixture, reaching essentially 100% at the highest temperatures. Third, upon returning to 190 °C reaction temperature, the Ru^0^ content decreases again, indicative of a slight reoxidation of the catalyst. The resulting level of about 90% metallic Ru^0^ closely resembles that obtained in CO_2_‐ref reformate, while during the TPR procedure the fraction is significantly higher than in CO_2_‐ref reformate.

Obviously, there is a distinct correlation between Ru reduction and CO_*x*_ methanation activity. Most clearly, this is demonstrated by the similar trends in the initial reaction phase at 190 °C (190‐C1). Hence, the initial activation at 190 °C is a consequence of the increasing reduction of the Ru NPs. Only the decay of the CH_4_ methanation rate in SR‐ref 6000 measured in the micro‐reactor (Figure 1a), after passing through the maximum, does not follow the trend in the metallic nature of the Ru NPs, which is about constant at later stages of the reaction (Figure 5). This discrepancy may be due to the different reaction conditions during the XAS measurement and in the microreactor. Alternatively, the deactivation observed in the kinetic measurement in Figure 1a may be due to the build‐up of reaction‐inhibiting adsorbed species such as CO_ad_ on the catalyst surface. Also, over‐reduction of the catalyst may be responsible for the deactivation in SR‐ref 6000, while in CO_2_‐ref, such effects should be less important.

Essentially identical Ru K‐edge XANES spectra were recently reported by Cisneros et al. during reaction on a high‐surface area Ru/TiO_2_ catalyst (235 m^2^ g^−1^) in SR‐ref 6000 at 190 °C^[^ ^31^ ^]^ under steady‐state conditions, after identical pretreatment. Also in that case, the Ru NPs were almost completely reduced to metallic Ru^0^. A complete profile over the same pretreatment and reaction sequence as in Figure 5 was also reported for a Ru/ZrO_2_ catalyst, for reaction in a SR‐ref 6000 gas mixture and under identical reaction conditions.^[^ ^32^ ^]^ These data are essentially identical to those presented in the bottom panel in Figure 5, with a rapid reduction of the Ru NPs, a further reduction during the TPR sequence, and a slight re‐oxidation upon returning to 190 °C. Evidently, the reduction of the Ru NPs during reaction occurs largely independent of the nature of the oxide support and their surface area in the reaction gas mixture, at least in the limited range presented here, and a largely metallic state of the Ru NPs is imperative for the CO_2_ methanation activity of these Ru catalysts.

In summary, these Ru K‐edge XANES data revealed a clear difference in the time required for essentially complete reduction of the Ru NPs, with about 350 min in CO_2_‐ref reformate and about 100 min in the presence of small amounts of CO in an otherwise similar CO_2_/H_2_ gas mixture (SR‐ref 6000). This agrees very well with the trends in the activation phases observed in the methane formation activity in Figure 1a, indicating a close correlation between the Ru reduction and catalyst activation. The Ti K‐edge XANES spectra further underline that also reaction in CO_2_/H_2_ at 190 °C, in the absence of CO in the atmosphere, results in a measurable presence of surface O vacancies on this catalyst.

### 2.3 Reaction Induced Structural Changes of Ru nanoparticles

In this section, we focus on the analysis of the structural properties of the Ru/TiO_2_ catalyst, specifically on the size and shape of the Ru nanoparticles at different stages of the CO/CO_2_ methanation reaction and at different temperatures, employing operando EXAFS measurements at the Ru K‐edge and a particle shape/size analysis based on post mortem TEM imaging. In the particle shape analysis, we follow an approach developed previously by our group earlier.^[^ ^15^, ^17^, ^29^ ^]^

**Figure 6:**
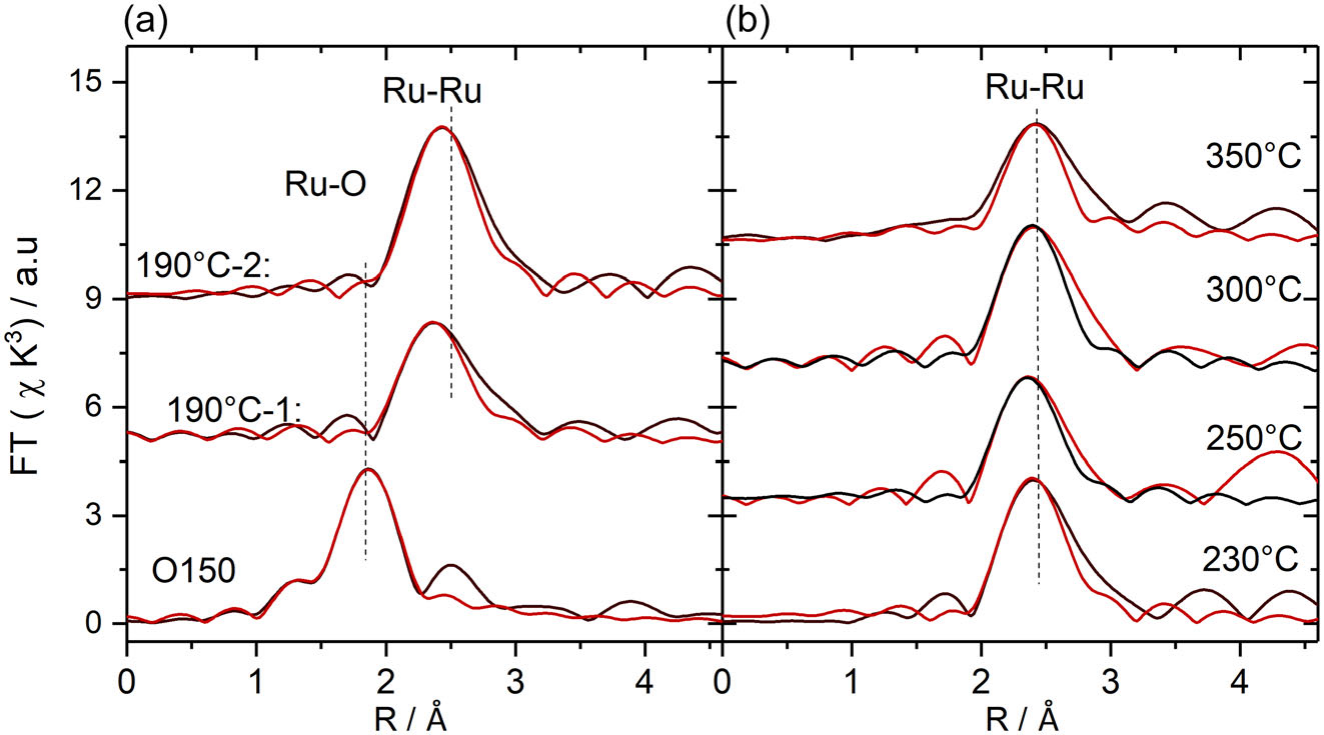
a) Representative Fourier transformed EXAFS functions derived from EXAFS measurements at the Ru K‐edge at 150 °C after calcination and change to N_2_ (O150), during reaction in SR‐ref 6000 gas mixture at 190 °C (300 min on stream) (190 °C‐1) and during reaction at 190 °C, 60 min after the TPR sequence (190 °C‐2). b) Similar data determined from measurements at different temperatures during the TPR sequence (230, 250, 300, and 350 °C) in SR‐ref 6000 (black: experiment, red: fit). The EXAFS data were collected together with the XANES data in Figure 5; each measurement took 3 min.

EXAFS data were recorded after the O150 activation step and during subsequent CO/CO_2_ methanation in SR‐ref 6000 reformate (see Table S1, Supporting Information). Representative Fourier transforms of the EXAFS spectra and their fits are shown in **Figure** **6**. The evaluation of the EXAFS spectra and the determination of the fit parameters, which follows the procedures used in earlier studies,^[^ ^17^, ^30^ ^]^ are described in the Experimental Section; the corresponding fit parameters are summarized in Table S1 in the Supporting Information.

After calcination, the Ru/TiO_2_ exhibited a significant contribution from a Ru—O coordination shell with a distance of 2.12 ± 0.04 Å and a Ru—O coordination number (CN_Ru—O_) of 1.5 ± 0.4. Upon switching to the reaction gas, this signal declined rapidly and was negligible already in the first spectrum (190 °C‐1, 10 min). At the same time, a maximum related to a Ru_—_Ru coordination shell appeared, reaching a CN_Ru—Ru_, of 3.0 ± 0.8. Considering the Ru–Ru distance of 2.62 ± 0.04, this signal is characteristic of the formation of very small metallic Ru nanoparticles. After 300 min on stream in the 190 °C‐1 phase, the particles had grown to a CN_Ru—Ru_ value of 6.4 ± 0.7. Assuming hemispherical shapes, this corresponds to a particle size of 1.6 ± 0.3 nm. Upon stepwise increasing the temperature from 190 to 350 °C, there seems to be a small decrease in Ru—Ru coordination number, with values around 5.5 on average. Considering the scatter of the data and the fact that after the TPR sequence the coordination number is also at 5.9, we conclude that Ru particle growth occurs mainly during the isothermal reaction at 190 °C, in the first 300 min, while the resulting particle size is constant both during the TPR treatment and after cool‐down, during subsequent reaction at 190 °C in the 190 °C‐2 phase.

**Figure 7:**
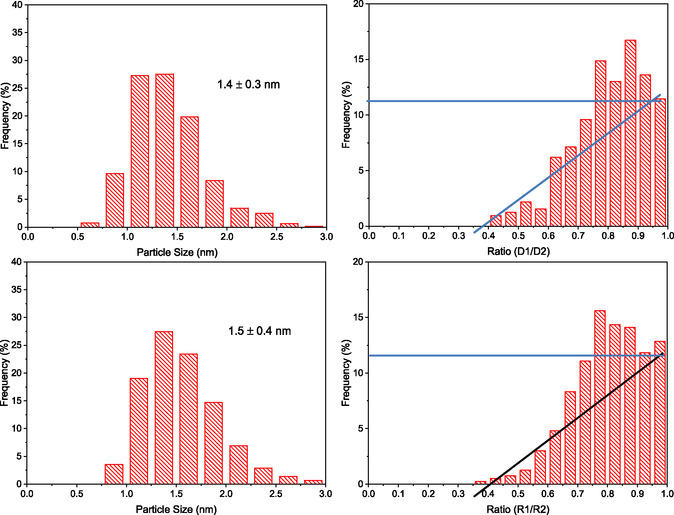
Particle size distributions (left panels) and particle shape distributions (ratio of the shorter diameter to the longer diameter) (right panels) evaluated from high‐resolution TEM images recorded after isothermal reaction in SR‐ref 6000 at 190 °C (upper panels) and after TPR treatment and subsequent isothermal reaction at 190 °C (lower panels).

Before comparing these trends with previous results, we will briefly present and discuss the results of an ex situ (post mortem) TEM‐based analysis of the Ru particle sizes and shapes after isothermal reaction in SR‐ref 6000, after 1000 min on stream in the 190 °C‐1 phase, and after the TPR sequence and 180 min on stream in the 190 °C‐2 phase. The resulting particle size and particle shape distributions are presented in **Figure** **7**. Comparison of the particle size distributions before and after the TPR sequence clearly confirms the trend derived from the EXAFS measurements that Ru particle growth is finished during the isothermal reaction at 190 °C and that the TPR procedure does not induce any further significant growth of the Ru nanoparticles. Even the absolute values of the Ru particle sizes are very close to those derived from the EXAFS measurements.

A possible flattening of the Ru nanoparticles can be identified from the ratio of the shorter diameter to the longer diameter in the projections of the Ru nanoparticles in TEM images. For spherical particles, this ratio would always be 1, while for hemispherical particles, we would expect ratios between 0.5 and 1.0, depending on the orientation of the nanoparticle.^[^ ^15^, ^17^ ^]^ For even flatter NPs, the lowest ratio should even be lower. Thus, the degree of the Ru particle flatness and changes therein can be qualitatively probed from these ratios, and a plot of this ratio for each of the NPs will provide qualitative information on the flatness of the NPs. Furthermore, any shift to lower ratios during the TPR treatments would be indicative of a TPR‐induced flattening of the nanoparticles. The data presented in the right panels in Figure 6 indicate that on average, the particles are slightly flatter than hemispherical particles. Furthermore, this distribution is rather close to the distribution obtained previously for a similar type Ru/TiO_2_ catalyst (P90 TiO_2_ support, similar Ru loading) after 1000 min on stream in SR‐ref 6000.^[^ ^15^ ^]^ In that work, we could show that the distribution reaches lower values as compared to comparable Ru/ZrO_2_ and Ru/Al_2_O_3_ catalysts, indicating that there is a more pronounced tendency for Ru NP flattening for the Ru/TiO_2_ catalyst than for the other ones. We had explained this flattening by a more pronounced tendency for O‐vacancy formation on TiO_2_ during exposure to the reductive gas mixture as compared to the other oxide support surfaces, which in turn leads to a stronger metal‐support interaction.^[^ ^15^ ^]^ Such kind of flattening seems to occur also in the present case, although it cannot be quantified on an absolute scale. Comparison with the distribution obtained after the TPR treatment indicates that there is no significant further TPR‐induced flattening of the Ru NPs.

The main results obtained for particle sizes and shapes after isothermal reaction (mean diameter 1.4 nm, particle shapes slightly flatter than hemispherical) can be compared with earlier results obtained for the same catalyst in a CO/H_2_ gas mixture (0.6% CO/3% N_2_/balance H_2_) at 190 °C.^[^ ^17^ ^]^ In that case, we obtained a rather similar particle shape distribution after 10 min on stream, while after longer reaction, 1000 min on stream, a distribution with a more pronounced maximum at a ratio of 0.8 was obtained.^[^ ^17^ ^]^ Apparently, in that more reductive gas mixture, structural modifications such as Ru NP flattening are more pronounced than in the SR‐ref 6000 gas mixture, which, in addition to some CO, also contains large amount of CO_2_. Finally, the general trend of little modification in the Ru particle size upon high‐temperature treatment in the SR‐ref 6000 gas mixture agrees very well with our earlier finding of no significant change in particle size upon a TPR treatment for a similar Ru/TiO_2_ catalyst in a CO_2_‐ref gas mixture (catalyst 2 in^[^ ^14^ ^]^).

In total, these results demonstrate that the interaction of the present, P90‐TiO_2_‐based Ru/TiO_2_ catalyst with a SR‐ref 6000 gas mixture under reaction conditions (190 °C) results in the rapid formation of very small metallic Ru^0^ NPs with a mean particle size of 1.5 nm, which is reached already after 300 min on stream. A high‐temperature treatment up to 350 °C in that reaction atmosphere does not lead to further particle growth, neither in CO_2_‐ref^[^ ^14^ ^]^ nor in SR‐ref 6000 reformate (recent work). O‐vacancy formation during exposure to the reaction gas results in stronger metal‐support interactions, leading to the formation of Ru particle shapes slightly flatter than hemispherical under reaction conditions, with no significant modification upon the TPR treatment.

## 3 Conclusions

Combining results of operando X‐ray absorption measurements with kinetic and electron microscopy data, we could show that the activation of highly active and selective CO_*x*_ methanation Ru/TiO_2_ catalysts in pure CO_2_/H_2_ reaction gas mixture (CO_2_‐ref) or in a mixture containing also small amounts of CO (SR‐ref 6000) is closely correlated with the reduction of the RuO_*x*_ nanoparticles. In the presence of small amounts of CO in the otherwise similar CO_2_/H_2_ gas mixture, activation and reduction are significantly faster than in the pure CO_2_/H_2_ gas mixture. A subsequent high‐temperature treatment up to 350 °C in the reaction gas results in a significant increase in the reaction rate at 190 °C. The activation results in the formation of very small (1.5 ± 0.4 Å diameter), whose size does not change measurably during longer reaction or upon high‐temperature treatment. According to a TEM‐based particle shape analysis, the Ru nanoparticles are slightly flatter than hemispherical, and maintain this shape also during high‐temperature treatment. This particle flattening is attributed to a rather strong interaction of the Ru nanoparticles with the partly surface‐reduced TiO_2_ support, caused by the formation of O‐vacancies. Finally, comparison with data reported for Ru catalysts supported on other oxides, both other types of TiO_2_, but also ZrO_2_ and Al_2_O_3_, indicates that this behavior is characteristic for oxide‐supported Ru nanoparticle catalysts with very small Ru particle sizes, which are of high technical relevance because of their high activity and selectivity in the Sabatier reaction, supported on reducible oxides.

## 4 Experimental Section

4.1

4.1.1

##### Catalyst Preparation and Pretreatment

The catalyst was prepared by an incipient wetness impregnation procedure, using commercially available TiO_2_ (P90, Evonik AG). Briefly, 63.5 mg of the Ru precursor (RuCl_3_ hydrate, Sigma Aldrich, 99.99%) were dissolved in 1.58 mL of Millipore water. Afterwards, 1 g of the corresponding support material (as received) was added under continuous stirring for 1 h at a rotation rate of 250 rpm, to ensure a homogeneous distribution of the Ru precursor on the oxide support. The resulting wet powders were subsequently dried at 25 °C overnight and then stored in a fridge until use. Prior to all experiments, the catalyst was pretreated in situ by calcination in a flow of 10% O_2_ in N_2_ (41.6 Nml min^−1^) for 30 min at 150 °C to remove residual chlorine impurities and to reduce the amount of carbon‐containing species. Using this procedure, we were able to reduce the amount of chloride anions significantly below 0.5% (based on surface atomic concentration derived from XPS). Additional investigations using EDX showed no signal for chloride (see previous discussion in^[^ ^13^, ^17^ ^]^). Subsequently, the catalyst was purged in a flow of N_2_ (41.6 Nml min^−1^) for 15 min at this temperature. Finally, the Ru catalyst was heated up in 5 min from 150 °C to the initial reaction temperature of 190 °C in the reaction gas.

##### Kinetic Measurements

Kinetic measurements were performed in a tubular flow microreactor (inner diameter 4.5 mm) at atmospheric pressure in a pure CO_2_/H_2_ gas mixture (CO_2_‐ref: 15.5 vol% CO_2_, 3 vol% N_2_, balance H_2_) or in a CO_2_/H_2_ gas mixture containing small amounts of CO (SR‐ref 6000: 15.5 vol% CO_2,_ 0.6 vol% CO, 3 vol% N_2_, balance H_2_). To achieve differential reaction conditions, the catalyst was diluted with α‐Al_2_O_3_, which was found to be inactive under present reaction conditions. About 200 mg of diluted catalyst (dilution 1:10) was located in the middle of the reactor, fixed by two pieces of quartz wool. This results in a catalyst bed of ≈1.2 cm length. After calcination at 150 °C and subsequent reduction of the catalyst, its activity was determined during 1000 min reaction at 190 °C, followed by a TPR sequence (stepwise at 210, 230, 250, 270, 300, and 350 °C, 3 h at each temperature). Finally, the catalyst was cooled down to 190 °C, followed by another isothermal reaction measurement at that temperature. Detailed descriptions of the experimental procedure and of the evaluation of the kinetic data were given in our related previous publications.^[^ ^11^, ^14^, ^29^ ^]^

##### Catalyst Characterization

The chemical composition of the catalyst was characterized by operando near‐edge X‐ray absorption spectroscopy (XANES) at the Ru K‐edge and at the Ti K‐edge. Structural characterization was performed under reaction conditions, by operando extended fine structure X‐ray absorption spectroscopy (EXAFS) and by post mortem (S)TEM imaging.

##### XAS Measurements

Time‐resolved operando XANES and EXAFS measurements were performed at different synchrotrons, using a specially designed reaction cell.^[^ ^18^, ^33^ ^]^ In these measurements, the amount of catalyst is roughly 2 times larger than the amount used in the kinetic measurements to allow for reasonable signal‐to‐noise ratio.

XANES measurements at the Ti K‐edge (4966 eV) were performed at the XAFS beamline of the electron storage ring Elettra in Trieste (Italy), using a Si(111) double crystal monochromator. Data were collected in the fluorescence mode, using a Si drift diode detector (Ketek GmbH, AXAS‐M, München, Germany). Since there was no possibility to simultaneously collect the signal of a reference (the sample was fully absorbing), Ti metal foil spectra were collected in between series of measurements to check possible energy drifts of the monochromator. All data were corrected for the dead‐time of the detector and for self‐absorption effects based on the self‐absorption procedure implemented in Athena (version 0.9.26).^[^ ^34^ ^]^ The contribution of the edge jump to the pre‐edge structure was accounted for by subtracting a background function (combination of a linear and a Lorentzian function).^[^ ^34^ ^]^ The analysis of the pre‐edge region was then performed by fitting a set of pseudo‐Voigt functions to the pre‐edge spectral envelope, using roughly the same FWHM values for all components.

Both the pretreatment and the reaction measurements were performed under operando conditions, including a simultaneous analysis of the gas outlet from the XAS cell. In these measurements, the reaction cell was loaded with 75 mg of diluted Ru/TiO_2_ catalyst (3:1 diluted with Al_2_O_3_). Reactants and products leaving the reaction cell during CO_2_ reduction (15.0 vol% CO_2_, 75.0 vol% H_2_, balance N_2_) were monitored by a home‐built analysis system consisting of a Bruker Alpha single‐beam transmission IR‐spectrometer (Ettlingen, Germany) and a substrate‐integrated hollow waveguide (iHWG) device for signal enhancement.^[^ ^35^ ^]^

Time‐resolved operando XAS measurements at the Ru K‐edge (22 117 eV) were performed at the energy‐dispersive ID24 beamline at the European Synchrotron Radiation Facility (ESRF), Grenoble. All XAS measurements were taken in transmission using a Si (111) polychromator in Laue configuration and utilizing a two‐dimensional FReLoN CCD detector.^[^ ^36^ ^]^ For these measurements, about 30 mg of diluted Ru/TiO_2_ catalyst (2:1 diluted with SiO_2_) was loaded in the reaction cell. The data acquisition took 10 s per spectrum. For the evaluation of the EXAFS data, we averaged the spectra collected over 15 min. A Ru foil, a pellet of Ru(IV) oxide, and RuCl_3_, which were measured in transmission mode, were used as reference materials for the data evaluation. Background removal and spectra normalization, as well as the LCA of the XANES spectra, were performed using the Athena software from the IFEFFIT program package.

Background subtraction and spectra normalization, as well as the LCA of the XANES spectra, were performed using the Athena software from the IFEFFIT program package.^[^ ^34^, ^37^ ^]^ On the basis of the linear combination analysis of the XANES spectra, the fraction of oxidized/reduced Ru nanoparticles was obtained by determining the contribution of the respective reference spectra to the catalyst/reaction spectra.^[^ ^37^, ^38^ ^]^ The fits displayed in the Supporting Information, cf. Figure S1 and S3, Supporting Information, include fits from the Ru(IV) oxide and RuCl_3_ separately; however, the fit itself is not sensitive to the different oxidic species, as one can see by the frequent changes/jumps in intensity between both species. Thus, we refer to oxidic species in the following.

The data reduction and subsequent fits of EXAFS spectra were carried out using the XDAP software package with standard procedures described elsewhere.^[^ ^39^ ^]^ Theoretical references were calculated by FEFF 8.0 and calibrated with experimental references of Ru foil and RuO_2_ powder.^[^ ^40^, ^41^ ^]^ The EXAFS data were evaluated in the R‐space (R: 0.0−4.3 Å), using the k‐range from 3.2 to 11.8 Å^−1^. In the EXAFS data fit, we allowed the coordination number (CN), the parameter σ in the Debye‐Waller factor (DWF), the Ru—Ru bond length (R), and the energy shift (*E*_0_) to change freely (see Table S1 in the Supporting Information). In most cases, the Ru—*O*—Ru scattering contribution was neglected in the EXAFS analysis due to negligible contribution from oxidic Ru species during the reaction. The amplitude reduction factor (S_0_^2^) used in the FEFF simulation of the EXAFS data was determined to be 0.95 for both RuO_2_ and Ru foil reference materials. This value was obtained by fitting the experimental reference spectra using FEFF Code‐generated models.

##### High‐Resolution (Scanning) Transmission Electron Microscopy (HR‐(S)TEM) Measurements

The Ru particle shape and size of the Ru/TiO_2_ catalyst were determined from bright field TEM and high‐angle annular dark‐field scanning transmission electron microscopy (HADDF‐STEM) measurements, which were performed on a Cs‐corrected FEI Titan electron microscope operated at 300 keV. For detailed information on the Ru particle size (volume‐area mean diameter and size distribution) and Ru particle shape (hemispherical and flat), at least 600 particles were evaluated for each sample. Assuming hemispherical Ru nanoparticles and a surface density of 1.5 × 10^15^ Ru atoms cm^−2^, the Ru dispersion was calculated from the volume‐area mean diameter.

With the known diameter (*d*_i_) of the individual Ru nanoparticles (*n*_i_), as measured by (S)TEM, the volume‐area mean diameter (*d*_VA_) was calculated according to Equation (1). From this relation one can easily calculate the Ru metal dispersion (*D*_Ru_), which is defined by the ratio of surface atoms to the total number of atoms in the hemispherical metal particle (*V*_Ru_ = volume Ru atom, *a*_Ru_ = surface area per Ru atom) as shown in Equation (2).
(1)

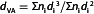

(2) $$D_{\text{Ru}}=6\;V_{\text{Ru}}/a_{\text{Ru}}d_{\text{VA}}$$

## Conflict of Interest

The authors declare no conflict of interest.

## Author Contributions

**Joachim Bansmann**: data curation (lead); investigation (equal); validation (equal); visualization (lead); writing—review and editing (equal). **Shilong Chen**: conceptualization: supporting; formal analysis (equal); investigation (equal); writing—original draft (supporting). **Ali M. Abdel‐Mageed**: conceptualization (equal); data curation (equal); investigation (equal); methodology (equal); supervision (equal); writing—original draft (equal). **R. Jürgen Behm**: conceptualization (lead); supervision (lead); writing—review and editing (lead).

## Supporting information

Supplementary Material

## Data Availability

The data that support the findings of this study are available from the corresponding author upon reasonable request.
