# Supplementary material for: Operando Ru and Ti K‐Edge X‐Ray Absorption Study of the Low‐Temperature Sabatier Reaction on Ru/TiO2 Catalysts
Source: Chemphyschem. 2025 Dec 17;27(1):e202500397. doi: 10.1002/cphc.202500397 (PMC12810640; doi:10.1002/cphc.202500397)
Supplement: Supplementary file 1 — Supplementary Material [file CPHC-27-e202500397-s001.pdf]

Supporting Information

Operando Ru and Ti K-edge X-ray Absorption Study of the Low-Temperature Sabatier Reaction on Ru/TiO_2 Catalysts

Joachim Bansmann,[1,] Shilong Chen[1,2,5], Ali M. Abdel-Mageed,[1,3*] R. Jürgen Behm[1,4*]*

[1]*Institute of Surface Chemistry and Catalysis, Ulm University, D-89069 Ulm, Germany*

[2]*Institute of Inorganic Chemistry, Christian-Albrechts-University, D-24118 Kiel, Germany*

[3]*Leibniz-Institut for Catalysis (LIKAT), D-18059, Rostock, Germany*

[4] *Institute of Theoretical Chemistry, Ulm University, D-89069 Ulm, Germany*

[5] *now: National Engineering Research Center of Chemical Fertilizer Catalyst,*

School of Chemical Engineering, Fuzhou University, 350002 Fuzhou, China

:

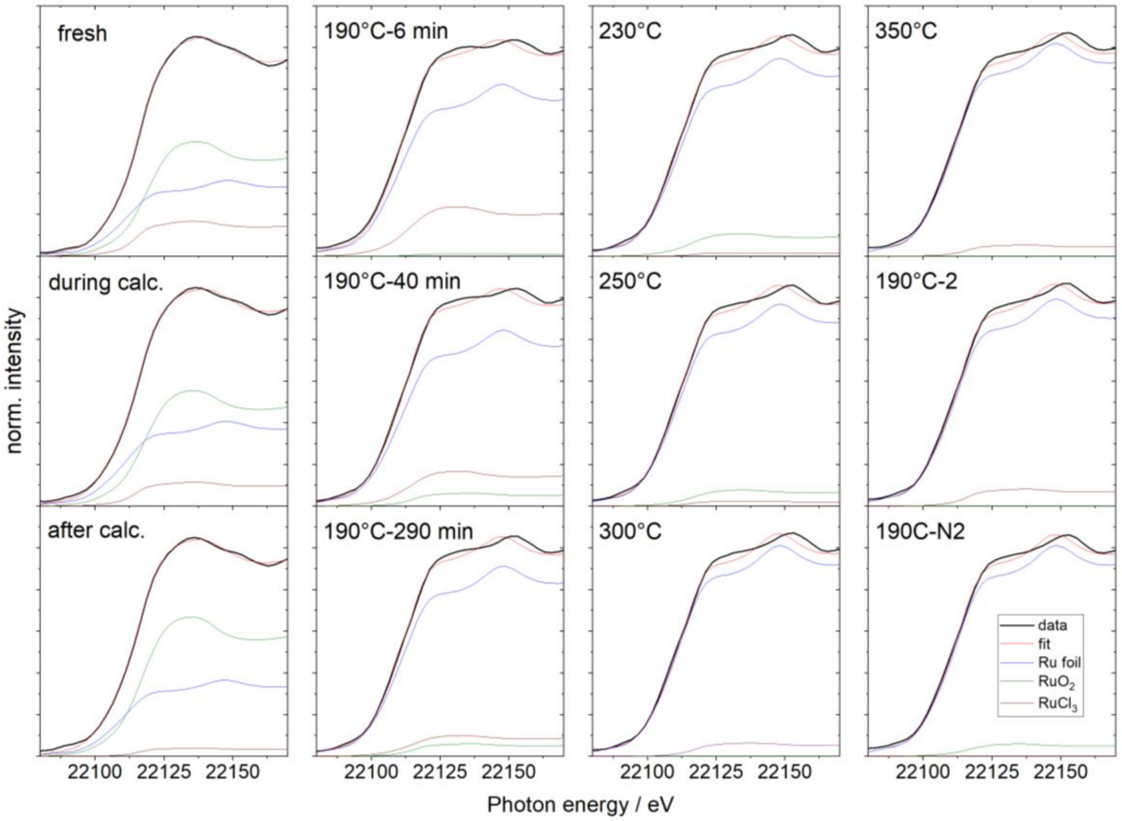

Figure S1: XANES spectra taken from Ru/TiO_2 catalysts at the Ru K-edge during subsequent steps of the CO_2 methanation reaction. Panels from left to right: 1st panel: before/during and after calcination, 2nd panel: during first reaction period (C-1) at 190 °C, 3rd panel: during TPR sequence from 230 to 300 °C, right panel, 4th panel: at final step of TPR sequence at 350°C and during second reaction period (C-2) at 190 °C and finally after reaction in N_2 at 190°C. Black: experimental data, red: fit; blue: Ru^0 contribution, green: RuO_2 contribution, brown: $RuCl_3$ contribution.

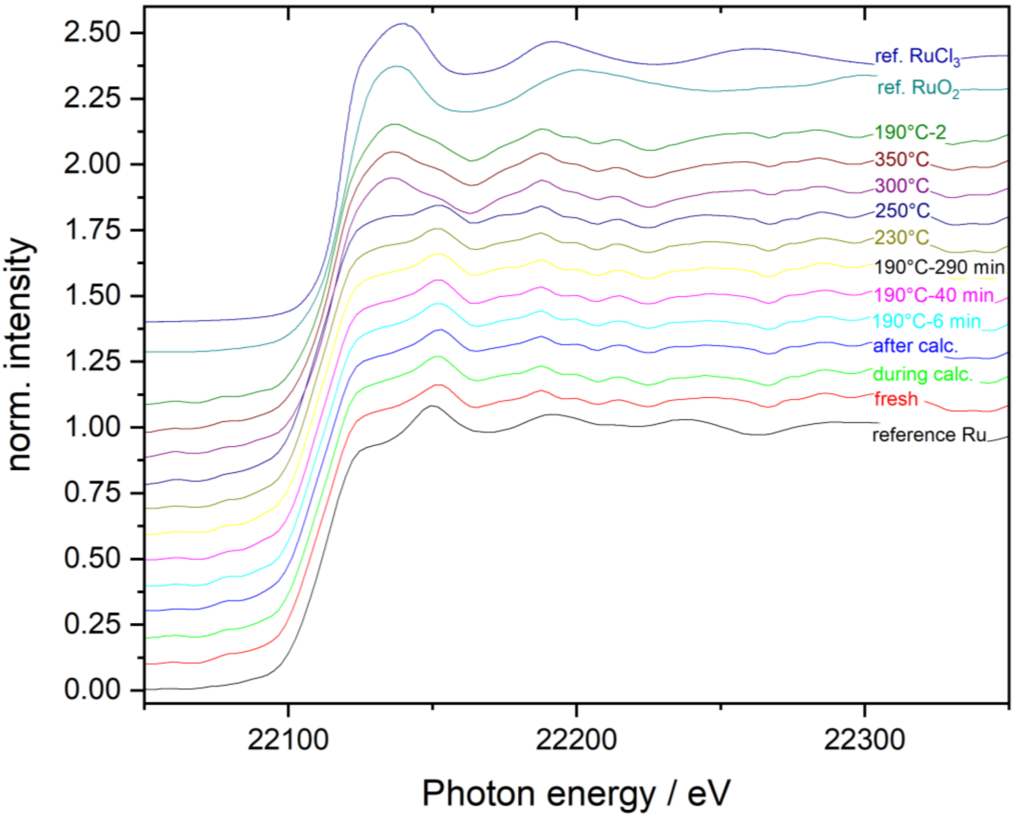

Figure S2: XANES spectra (raw data) taken from Ru/TiO_2 catalysts and Ru references at the Ru K-edge during subsequent steps of the CO_2 methanation reaction.

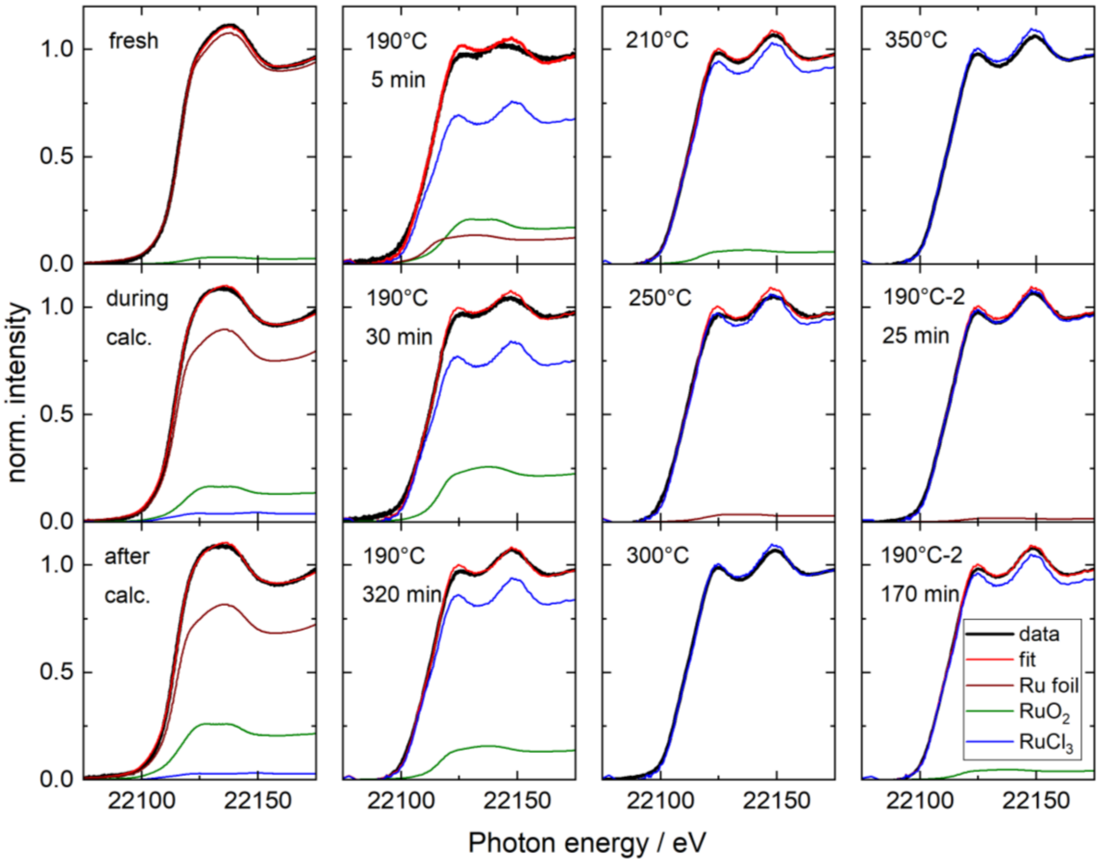

Figure S3: XANES spectra taken from Ru/TiO_2 catalysts at the Ru K-edge during subsequent steps of the CO methanation reaction. Panels from left to right: 1st panel: before/during and after calcination, 2nd panel: during first reaction period (C-1) at 190 °C, 3rd panel: during TPR sequence from 230 to 300 °C, right panel, 4th panel: at final step of TPR sequence at 350°C and during second reaction period (C-2) at 190 °C. Black: experimental data, red: fit; blue: Ru^0 contribution, green: RuO_2 contribution, brown: $RuCl_3$ contribution.

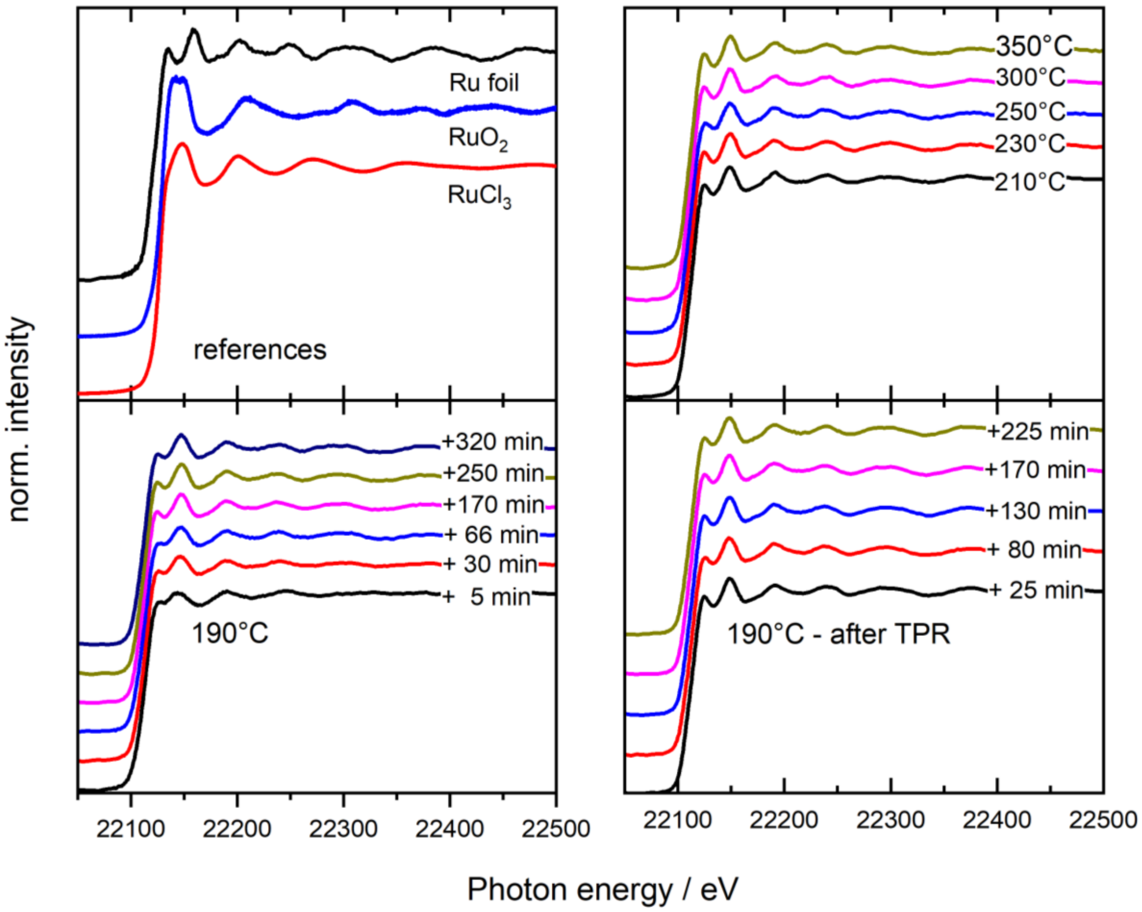

Figure S4: XANES spectra (raw data) taken from Ru/TiO_2 catalysts and Ru references at the Ru K-edge during subsequent steps of the CO methanation reaction. Left part: references (top) and during reaction at 190°C (bottom), right: during TPR sequence (top) and during second reaction part (190°C-2) after TPR (bottom).

Table S1: Fit parameters for the EXAFS spectra collected after calcination (O150) in N_2 at 150 °C and during the subsequent CO/CO_2 methanation reaction in SR-ref 6000 gas mixture at different temperatures.

State of the catalyst	$R_{Ru\text{-}O}$, $R_{Ru\text{-}Ru}$ / Å	$CN_{Ru\text{-}O}$ / $CN_{Ru\text{-}Ru}$	DWF(Ru-O/Ru-Ru) / Å^2	E_0 / eV
After O150 calcination, at 150 °C	2.12 ± 0.04	1.5 ± 0.4	0.014± 0.006	-7 ± 0.5
At 190°C-1, 10 min	2.23 ± 0.02<br>2.62 ± 0.04	0.8 ± 0.5<br>3.0 ± 0.8	0.004 ± 0.01<br>0.01 ± 0.01	-7 ± 3<br>11 ± 3
At 190°C-1, 100 min	2.66 ± 0.02	4.1 ± 0.7	0.005 ± 0.003	5 ± 1.2
At 190°C-1, 300 min	2.67 ± 0.02	6.4 ± 0.7	0.006 ± 0.002	9.2 ± 1
At 230°C	2.67 ± 0.02	5.9 ± 0.8	0.007 ± 0.003	7 ± 1
At 250°C	2.64 ± 0.02	5.3 ± 0.8	0.007 ± 0.003	6.3 ± 1.1
At 300°C	2.65 ± 0.02	5.9 ± 0.8	0.007 ± 0.003	2.2 ± 1
At 350°C	2.65 ± 0.02	5.2 ± 0.8	0.008 ± 0.003	4.3 ± 0.4
At 190°C-2	2.67 ± 0.02	5.9 ± 0.7	0.005 ± 0.002	8 ± 1

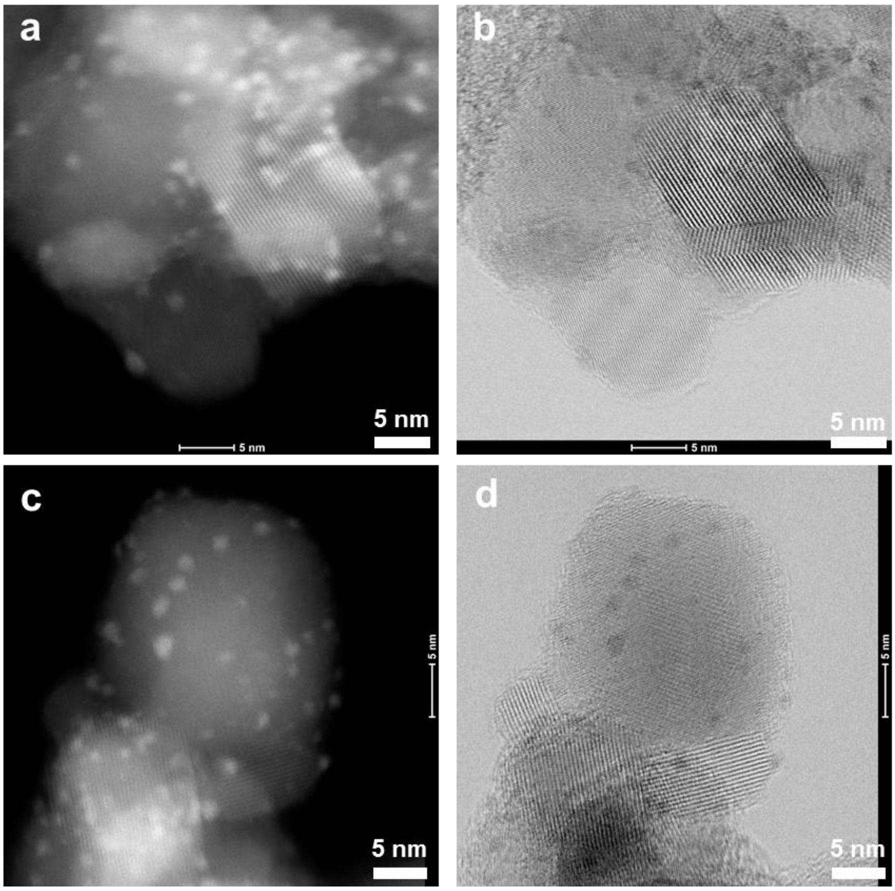

Fig. S5. The STEM images of Ru/P90 after isothermal reaction at 190 °C in CO_2-ref: (a, c) Dark field images, (b, d) the corresponding bright field images.

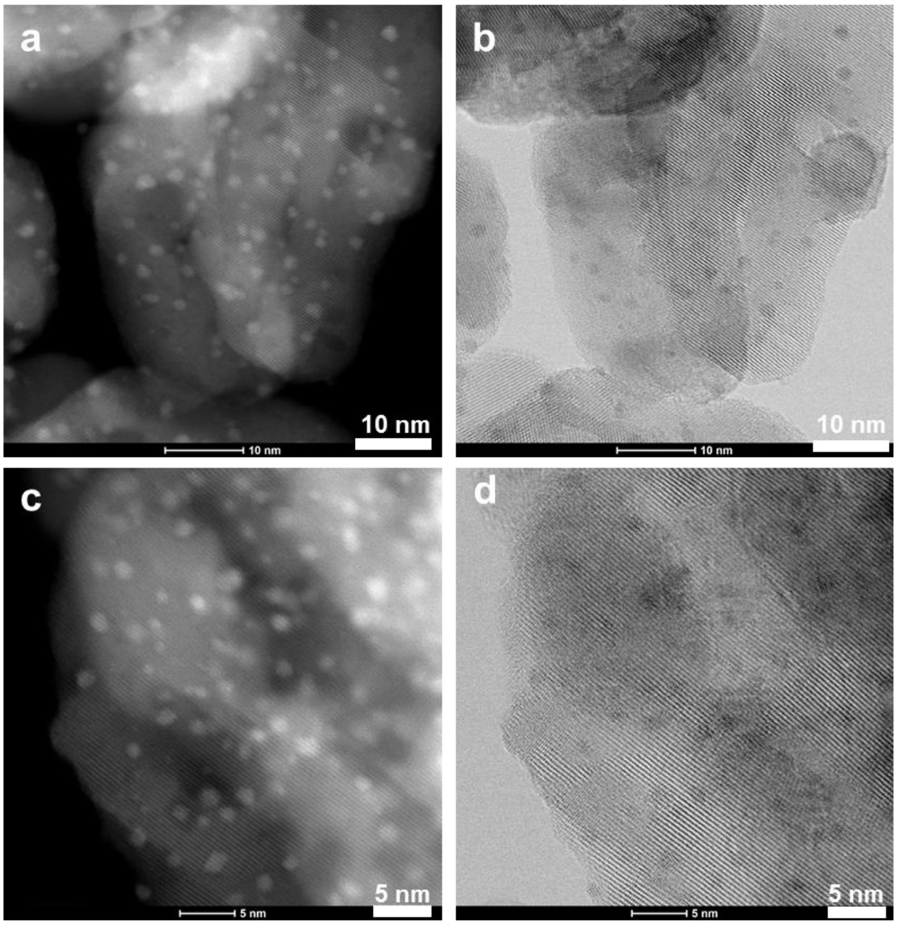

Fig. S5. The STEM images of Ru/P90 after TPR in CO_2-ref: (a, c) Dark field images, (b, d) the corresponding bright field images.
